# Non-seed plant research in the spotlight

**DOI:** 10.1242/bio.062139

**Published:** 2025-12-09

**Authors:** Karima El Mahboubi, Facundo Romani

**Affiliations:** ^1^ Laboratoire de Recherche en Sciences Végétales (LRSV), Université de Toulouse, CNRS, UPS, Toulouse INP, Castanet-Tolosan 31320, France; ^2^ Department of Plant Sciences, University of Cambridge, Cambridge CB2 3EA, UK

**Keywords:** Non-seed plants, Evo-devo, Model organisms, Genomics

## Abstract

The Genetics Society Non-Seed Plant meeting brought together researchers embracing the diversity of plants and using emerging and established model systems covering hornworts, mosses, liverworts, lycophytes and ferns. This growing community of researchers is exploring fundamental questions on plant development, evolution and environmental responses. Highlights included cutting-edge work in bryophytes on meristem development, hormonal signalling and chromatin regulation, as well as advances in charophyte algae, illuminating the evolutionary origins of key plant traits. The meeting emphasized how non-seed plants, often overlooked in mainstream plant science, are now providing transformative insights into gene regulation, plant-environment interactions and crop improvement potential. These developments reflect a broader shift in plant biology, where diverse model systems are essential for reconstructing the evolutionary history of plants and addressing modern agricultural challenges.

## Teaser

**Summary:** With non-seed plant research gaining momentum and a vibrant community emerging, this Meeting Review highlights the growing impact of diverse model systems on key questions in plant evolution.

## Introduction

For decades, plant science research has predominantly focused on vascular plants, particularly angiosperms (flowering plants) and the model *Arabidopsis thaliana*. In recent years, together with the availability of new fully sequenced genomes, there has been a growing recognition of the need to expand model systems beyond angiosperms to address fundamental questions in plant biology and to unravel the diversity of plants (Rensing, 2017). Non-seed plants, including bryophytes and pteridophytes (lycophytes and monilophytes/ferns), hold key phylogenetic positions that are essential for bridging evolutionary gaps in our understanding of land plant evolution and their origin ∼470 million years ago (Morris et al., 2018). They shed light on into the origins of major innovations that enabled terrestrial adaptation and the diversification of land plants. Investigating plant evolution involves integrating evidence from the fossil record (Tomescu and Rothwell, 2022), morphological analyses (Jill Harrison, 2017) and molecular approaches (Delaux et al., 2019).

The number of researchers embracing non-seed plant research has grown significantly across the globe in the past decade. This includes the establishment of model organisms, such as the moss *Physcomitrium patens*, the liverwort *Marchantia polymorpha*, and emerging ones such as the hornwort *Anthoceros agrestis*, the lycophyte *Selaginella* sp. and the fern *Ceratopteris richardii.*

The building of community hubs is fundamental for the long-term growth of this field of plant research. Justin Goodrich (University of Edinburgh, UK) and Laura Moody (University of Oxford, UK) established the ‘non-seed plant’ special interest group with the support of the Genetics Society. Successive meetings in Oxford, Birmingham and Cambridge have been held every year since 2022. Rotating between different locations in the UK, the latest edition was held in Madingley Hall, Cambridge, bringing together researchers in the UK alongside a growing number of researchers from across Europe and the United States. The meeting places a strong emphasis on supporting early-career researchers (ECRs), who represent most of the speakers. Relying on the incredible diversity of speakers, model systems and topics, this meeting aims to promote resource sharing and foster collaborations. This Meeting Review highlights the latest trends in non-seed plant research and some of the recent findings presented at the meeting.

## Model organisms and the origin of land plants

Model organisms are fundamental to research by serving as simplified and tractable systems to study complex biological processes (Cesarino et al., 2020). To understand the origin of land plants (embryophytes), it is critical to have an extant model system sister to the rest (traditionally referred as basal). The journey in that direction for non-seed plants started with the moss *P. patens* (hereafter *Physcomitrella*), which emerged as the first widely recognized model system (Reski and Frank, 2005). The adoption of *Physcomitrella* was primarily driven by its rare ability among plants to undergo efficient homologous recombination, enabling precise genetic engineering. More recently, the liverwort *M. polymorpha* (hereafter Marchantia) has gained rapid popularity due to its simple cultivation, lack of whole-genome duplications and suitability for genetics (Bowman et al., 2022). Beyond land plants, significant progress has been made in developing new model systems and genomic resources for the charophycean algae, algal relatives that colonized land before the divergence of embryophytes (Kunz et al., 2025). However, there is still not a consolidated candidate, and genetic tools remain limited.

**Fig. 1.**
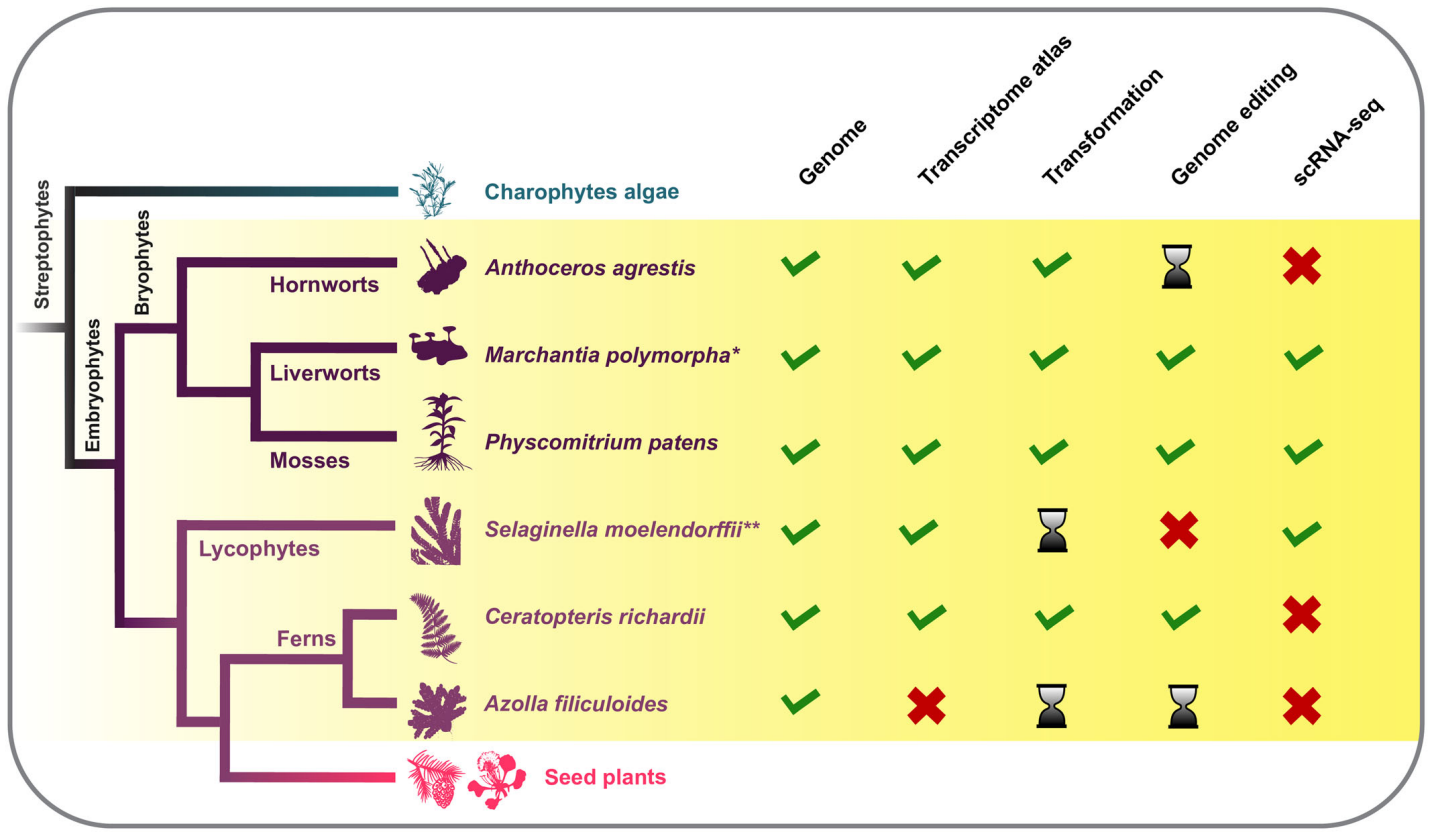
**Model systems and available resources in non-seed plants.** Overview of available and developing resources (sequenced genomes and transcriptomes, single-cell transcriptomics) and experimental methods (stable transformation, CRISPR-Cas-based genome editing) for the main models. *Marchantia paleacea could also be considered a model system for symbiosis and also has several resources. **Several tools were also developed for *Selaginella kraussiana*, and the single-cell RNA sequencing (scRNA-seq) is exclusive to *S. kraussiana*.

For decades, which clade is sister to the majority of land plants is a question that has sparked some discussions between proponents of different model organisms (Puttick et al., 2018). The rapid development and increasing accessibility of sequencing technologies have significantly expanded the repertoire of sequenced plant genomes and transcriptomes (One Thousand Plant Transcriptomes Initiative, 2019; Dong et al., 2025), as well as clarified the phylogenetic relationships between the different clades (Fig. 1). The prevalent model is that bryophytes (hornworts, mosses and liverworts) form a monophyletic group, with mosses and liverworts being sister clades (Donoghue et al., 2021). This has several implications, but probably the most important one is that none of the extant bryophyte is ancestral to either vascular plants or land plants.

The proliferation of different model systems has ultimately proven to be highly complementary, with each having different advantages and limitations (Naramoto et al., 2022). The usefulness of a model organism is associated with its genetic tractability, resources and tools, and not necessarily tied to its phylogenetic position or developmental features. In practice, the different model organisms provide useful insights as far as they are used for appropriate scientific questions. Other extant plant models (or not) are also key to achieving a complete picture of the evolutionary history of plants, including lycophytes, monilophytes and even fossil species. Embracing diversity is essential for exploring the common features shared by all land plants and the unique traits (synapomorphies) that define specific lineages. Research presented at the Non-Seed Plant meeting clearly reflects this.

## Emerging model systems to bridge the gaps in land plant research

Beyond well-established models, additional non-seed plant models have been developed to broaden the diversity of studied organisms or to investigate different traits. Monilophytes, including ferns and their relatives, with over 10,500 species, represent the second largest lineage of plants, following angiosperms (Marchant et al., 2022). Ferns share many characteristics with bryophytes, including reproduction via spores and an independent gametophyte generation, and with seed plants, such as vasculature and an independent sporophyte generation (Kinosian and Wolf, 2022). Therefore, they constitute a key evolutionary lineage, offering both a crucial reference point for understanding innovations in seed plants and an intermediate lineage to connect functional genomics across land plants.

In the mid-1960s, *C. richardii* (hereafter *Ceratopteris*) (Fig. 1) emerged as a fern model because of its relatively short life cycle for a fern (120 days) and its ease of cultivation under controlled conditions (Chatterjee and Roux, 2000). For decades, *Ceratopteris* served as a model to study developmental aspects, including sex determination, photomorphogenesis and rhizoid development (Kinosian and Wolf, 2022). However, the lack of a reference genome – delayed by challenges including the large genome size – and the absence of a genetic transformation system, have limited the exploration of fern biology. Nevertheless, recent advances including publication of the first reference genome (Marchant et al., 2019) and transcriptome (Geng et al., 2021), combined with the development of protocols for stable transformation (Plackett et al., 2014) and genome editing tools (Jiang et al., 2024), are unlocking the full potential of this model system. In the Non-Seed Plant meeting, Andrew Plackett presented his recent advances in building a comprehensive transcriptional expression atlas of the sporophytic and gametophytic generation of *Ceratopteris* (Plackett and Catoni, 2025 preprint).

The unique features of the life cycle of ferns offer numerous benefits, and the ability to generate *Ceratopteris* transgenic lines in both generations provides a unique perspective for studying sporophytes and gametophytes independently. However, *Ceratopteris* may not fully represent the diversity of ferns, highlighting the need for additional models. More recently, the publication of a comprehensive set of transcriptomes across ferns (Ali et al., 2025) will be key to embracing their diversity. The aquatic fern *Azolla filiculoides* (Fig. 1) has also emerged as a promising fern model for evolutionary developmental biology (evo-devo) studies, as it is a simple laboratory system and has one of the smallest genomes (750 Mb versus several Gb) among ferns (Li et al., 2018). *A. filiculoides* has also been developed as a model to study a unique symbiosis with the nitrogen-fixing cyanobacterium *Anabaena azollae*, which is essential for its growth (de Vries and de Vries, 2022). Thomas Torode's group (University of Keele, UK) is actively developing new resources for this model.

Alexander Hetherington (University of Edinburgh, UK) presented recent findings on the evolutionary origin of fern fiddleheads (early-stage fern fronds). Comparative studies of extant ferns identified fiddleheads as a synapomorphy of the circinatophytes clade and suggest that fiddleheads evolved alongside a transition from tetrahedral to wedge-shaped apical cells in the leaf meristems. Fossil evidence from the 315-million-year-old fern *Ankyropteris corrugata* revealed the presence of a tetrahedral apical cell in the leaf meristem, supporting this hypothesis (Cruz and Hetherington, 2025). These findings align with the telome theory, proposing that fern leaves evolved through the modification of shoots, and provide new insights into the evolution and origin of leaves in vascular plants – one of the longstanding questions in plant evo-devo. Hetherington's research exemplifies the potential of multidisciplinary approaches, including paleobotany, genomics and modern microscopy techniques, to answer fundamental questions in evo-devo (Hetherington, 2024).

Moving away from ferns, lycophytes (clubmosses, spikemosses, quillworts) represent another lineage exhibiting typical traits of both vascular and non-vascular plants, making them extraordinary systems for understanding the evo-devo of roots and meristems. The publication of the *Selaginella moellendorffii* genome in 2011 (Banks et al., 2011) marked a key milestone for non-seed plant research. However, essential tools and resources to investigate gene functions (e.g. gene editing, stable transformation) are still lacking. Despite these challenges, research on lycophytes development has made significant progress (Spencer et al., 2021). The application of spatial transcriptomics in *S. moellendorffii* (Yang et al., 2023) is a good example of how vibrant the research on this model system remains. Alternative *Selaginella* species, such as *Selaginella apoda* and *Selaginella kraussiana*, may hold the key to finally reveal the full potential of this model system (Spencer et al., 2023) by allowing genetic transformation. However, not everything is about *Selaginella*: Yuki Ito presented his work on root development in *Lycopodium clavatum* (Ito et al., 2022), and Ana Julia Sagasti discussed fossilized lycophytes from the Rhynie chert, such as *Asteroxylon mackiei* (Turner et al., 2023).

Another key lineage are hornworts, which hold the missing piece in the puzzle of bryophyte research. *Anthoceros* species (Fig. 1) have recently emerged as promising model species for experimental research (Frangedakis et al., 2021). *A. agrestis* displays typical characteristics of an ideal laboratory model, including easy cultivation under axenic conditions, a short life cycle with sporophyte induction within 1-2 months, ease of crossing and a non-duplicated genome, which simplifies functional analyses (Frangedakis et al., 2021). Among many interesting aspects of their biology, hornworts also (1) have larger sporophytes than mosses and liverworts, (2) engage in symbiosis with the nitrogen-fixing cyanobacteria and (3) present a carbon-concentrating mechanism (CCM).

Both algae and land plants (e.g. C_4_ and CAM species) use CCM to increase CO_2_ concentration around Rubisco. Pyrenoid-based CCMs (pCCMs) have been extensively studied in the green alga *Chlamydomonas reinhardtii*. Hornworts, as the only land plants to possess pCCMs, offer a unique opportunity to understand how CCMs may function in crops (Frangedakis et al., 2021). Fay-Wei Li (Boyce Thompson Institute, USA) presented recent advancements, showing that pyrenoids in *A. agrestis* and *C. reinhardtii* share liquid-like properties but differ structurally, lacking starch sheaths and being surrounded by multiple thylakoid membranes (Robison et al., 2025). Additionally, proteins required for algal pCCMs are present in *A. agrestis* and show similar subcellular localization. These findings suggest a conserved evolutionary origin of pCCMs in algae and hornworts, offering a new model for investigating pCCMs in land plants.

Emerging model organisms are not only limited to new lineages, but different species also have the potential to study traits that are absent in one model system. For example, *Marchantia paleacea* (Fig. 1) has emerged as an ideal model to study the evolution of arbuscular mycorrhizal (AM) symbiosis (Rich et al., 2021), the most widespread type of symbiosis. Unlike *M. polymorpha*, *M. paleacea* expresses genes essential for establishing AM symbiosis (Radhakrishnan et al., 2020). Anson Lam (John Innes Centre, UK) presented his recent work on calcium signalling in *M. paleacea* (Lam et al., 2024). Unlike in flowering plants, in *M. paleacea*, calcium signalling is only required for thallus colonization and not for penetration within rhizoids.

## Unravelling the evolution of genetic regulatory networks

Among land plants, meristem anatomy exhibits considerable diversity, and it is one of the major topics of evo-devo. In bryophytes, the dominant vegetative growth phase occurs during gametophytic generation, and the meristem presents important anatomical differences (Romani et al., 2024) from the sporophytic vegetative meristem present in vascular plants. Victoria Spencer (Gregor Mendel Institute, Austria) presented her work on the anatomy and branching patterning in Marchantia (Spencer et al., 2025). Under normal light conditions, Marchantia meristems undergo continuous dichotomous branching, maintaining symmetry between the bifurcated meristems. However, some meristems can become dormant under different light conditions (Streubel et al., 2023). Building on this research, they recently identified a cytochrome P450 enzyme specifically expressed in the active meristems, which inhibits meristem initiation and is required for promoting this asymmetry under far-red light conditions. This developmental role for a P450 could open new avenues to identify novel chemical morphogens involved in patterning in land plants.

More presentations demonstrated how Marchantia is now at the forefront of addressing major biological questions. Among them, James Walker (Salk Institute, USA) presented evidence that transcription-coupled DNA methylation, a mechanism long-studied in animals and flowering plants, is also active in Marchantia (Walker et al., 2025); Sarah Robinson (Sainsbury Laboratory Cambridge University, UK) presented her work on evolution of cell walls and cell shape (Bonfanti et al., 2023); and Bharti Aggarwal presented evidence on differential expression of miRNAs in Marchantia (Aggarwal et al., 2024). This emphasizes Marchantia's growing status as a genetically tractable model for cutting-edge questions.

*Physcomitrella* meristems are also an important source of novel discoveries. Developing apical cells with three or more cutting faces was crucial for plants to acquire the morphological complexity necessary for both land survival and the colonization of diverse ecosystems (Moody, 2022). As 3D growth happens early in embryonic development, it is challenging to investigate the shift from 2D to 3D growth in most plants. In the moss *P. patens*, the prolonged 2D filamentous development phase can be maintained indefinitely, removing the need to induce 3D growth (Moody et al., 2018). Taking advantage of this ideal model, Moody's group (University of Oxford, UK) has investigated 3D growth by studying the 2D to 3D transition. Through forward genetic approaches, they identified several *NO GAMETOPHORES* (*NOG*) genes involved in the transition to 3D growth in *Physcomitrella* (Moody, 2022). Katherine Mather, Gargi Chaturvedi and Rency Raquid introduced some of the newly identified *NOG* genes. *Physcomitrella* is a powerful model for genetic screening and for identifying novel genes that are specific to the gametophytic meristem or genes with roles largely unexplored in flowering plants.

Genetic networks regulating the apical meristem of bryophytes and vascular plants also display common features that are conserved across land plants. Jill Harrison (University of Bristol, UK), whose group aims to describe the developmental and genetic basis of shoot growth and branching (Harrison and Morris, 2018), presented their recent findings on the CLAVATA and WOX signalling pathways and their expression patterns in *Physcomitrella* (Nemec-Venza et al., 2025).

Beyond development, Philip Carella's group (John Innes Centre, UK) has been investigating the evolution of plant-microbe interactions in non-seed plants in challenging critical concepts. A common misconception is that bryophyte research cannot be applied to crops due to the long evolutionary distance. Among the recent advances shared by Carella's group was the discovery that diverse immunity factors found in non-flowering plants are transferable to flowering plants, offering a potential source of novel resistance genes against pathogens in crops (Chia et al., 2024). The ongoing development of pathogens and arbuscular mycorrhizal symbiosis systems (Lam et al., 2024) in non-seed plants is providing new insights into the evolution of plant-microbe interactions, with significant implications for both fundamental plant science and agricultural innovation. This includes pathogens isolated from wild Marchantia, such as *Pseudomonas viridiflava* (Robinson et al., 2025) and *Colletotrichum nymphaeae* (El Mahboubi et al., 2024 preprint), which allow us to explore of these interactions in a natural context.

## Beyond land plants

To understand the evolution of plants into the land environment, it is important to go beyond embryophytes and explore their closest aquatic ancestors. Charophytic algae offer that window and are providing fundamental clues to understanding plant development. Using multiple model systems and leveraging the growing number of sequenced genomes and transcriptomes, the Weijers group (University of Wageningen, The Netherlands) has significantly advanced our understanding of how the auxin signalling pathway evolved and complexified throughout plant evolution. Jorge Hernández-García presented their recent advances in algae and non-seed plants. Their findings showed that auxin and its precursor tryptophan trigger similar transcriptional responses in *Penium margaritaceum*, suggesting that the auxin signalling pathway became more complex during land plant evolution (Carrillo-Carrasco et al., 2025). Also, the investigation of the origin and evolution of auxin-responsive transcription factors (ARFs) revealed the existence of an ancestral class of ARF in algae, the ABC class. This class likely duplicated before diverging into the AB and C classes early in streptophytes. Additionally, heterologous complementation experiments with Marchantia suggest that a second event of duplication, in the AB class, occurred in the last common ancestor of land plants and gave rise to the A-ARF and B-ARF classes (Hernandez-Garcia et al., 2024). Evolution of essential traits followed this duplication and were essential for the rise of the nuclear auxin signalling pathway.

Finally, Bruno Catarino presented his work on the evolution of light and temperature integration across diverse plant models, using phenotypic and transcriptomic analyses. This work – including the models *M. polymorpha*, *A. thaliana*, the green algae (*Ostreococcus tauri*) and the streptophyte algae *Mesotaenium endlicherianum* – uncovers how gene regulatory networks change across hundreds of millions of years of evolution, including lineage-specific variations in the response to environmental cues, and shed light on the potential molecular mechanisms that drive these changes. Another example of comparative analysis of environmental responses featured across distant lineages in the conference was shared by Laura Delle Carbonare and Francesco Licausi (University of Oxford, UK). Their work involved the exploration of oxygen-sensing mechanisms across land plants (Dalle Carbonare et al., 2025).

## Collaborations are the key

By bringing together researchers, including ECRs, working on various aspects of plant biology and different models, this meeting has fostered collaborations and enabled comparative biology across distant plant lineages. A broad diversity of model organisms will strengthen the reconstruction of trait evolution across all plants (Delaux et al., 2019). Beyond the Genetics Society Non-Seed Plants Interest Group, other initiatives are making great progress too. In Germany, MAdLand (Molecular Adaptation to Land) investigates the genetic mechanisms of plant terrestrialization using diverse models, with a special focus on streptophyte algae and non-seed plants. In Spain, EvoDevoSigNet focuses on the evolution of developmental processes and signalling in plants. A growing community will continue playing a central role in shaping our understanding of plant evolution. We look forward to the next Non-Seed Plant meeting in December 2026 in Norwich.
